# Patient satisfaction level and its determinants after admission in public and private tertiary care hospitals in Bangladesh

**DOI:** 10.3389/frhs.2022.952221

**Published:** 2022-09-07

**Authors:** Farhana Begum, Jamaliah Said, Syed Zabid Hossain, Md. Ayub Ali

**Affiliations:** ^1^Ministry of Education, Dhaka, Bangladesh; ^2^Accounting Research Institute, Universiti Teknologi MARA, Shah Alam, Malaysia; ^3^Department of Accounting and Information Systems, University of Rajshahi, Rajshahi, Bangladesh; ^4^Department of Statistics, University of Rajshahi, Rajshahi, Bangladesh

**Keywords:** patient satisfaction, inpatient department, treatment cost, public hospital, private hospital

## Abstract

**Introduction:**

Patient satisfaction is an important indicator of the quality of care provided by health care facilities. The objective of this study was to investigate the rate of satisfaction and its associated factors among the patients admitted to tertiary care hospitals in Bangladesh.

**Methods:**

This cross-sectional study was conducted in a public and two private tertiary care hospitals in Bangladesh in December 2019, including 923 consecutive patients admitted to medical and surgical departments. Face-to-face interview using a structured questionnaire was conducted to collect patient-level data. Logistic regression models were used to determine the factors associated with patients' satisfaction.

**Results:**

Patients' overall satisfaction level was 65% (51% in public and 75% in private hospitals) with a satisfaction rate of 63% in hospital staff courtesy, 56.5% in a hospital environment, 67% in physician care, 63% in general patient satisfaction, and 58% in patient's family care. Private hospitals (aOR 3.64, 95% CI 2.2–6.03), conservative management (aOR 3.34, 95% CI 2.10–5.33), shorter hospital stay (aOR 1.58, 95% CI 1.05–2.37) and perceived improvement after treatment (aOR 1.67, 95% CI 1.01–2.76) were associated with patients' satisfaction. In contrast, patients' accommodation on the floor (aOR 0.38, aOR 0.22–0.66) and high health care costs (aOR 0.97, 95% CI 0.95–0.99) were associated with patients' dissatisfaction with the in-patient service they received in both public and private hospitals.

**Conclusion:**

Almost two-thirds of the patients were satisfied with the inpatient service they received, though, the satisfaction rate was higher in private hospitals. Treatment modality, cost, and outcome, as well as hospital environment like accommodation, were associated with their satisfaction level.

## Introduction

Patient satisfaction refers to their belief and an expression of attitude about the health care service they received. It depends on a number of components including expectations, service consumption experience, and experience-based emotional or cognitive response after consumption and choice. Hence it is a subjective evaluation of the patients' cognitive and emotional reactions resulting from the interaction between their expectations and perception of actual care received (1). Patient satisfaction has become an important indicator of the quality of care provided by health care facilities (2, 3). It is an essential component of convenient patient-centered care and plays a significant role in the health care delivery system. Dissatisfaction with healthcare services often results in a poor treatment outcome as these patients have an increased chance of missing appointments, non-adherence to treatment plans, and leaving hospitals against their advice (4). Despite this fact, it is reported that more than one-third of the patients are dissatisfied with the service they receive from different health care facilities (5).

A number of personal, socioeconomic, and facility-related factors are associated with the satisfaction of patients regarding health care delivery. Patients' characteristics like older age, higher educational and economic attainment, previous experience of hospital visit or admission, having multiple comorbidities, behavioral factors like higher expectations and negative attitude toward hospital service, and hospital-related factors like hospital size, location, environment, patient-staff ratio, communication with hospital staffs, etc. might shape the level of satisfaction of the patients (6–8).

Bangladesh, a developing country in the Southeast Asian region is well recognized for its shortage of a healthy workforce (9, 10). The quality of care provided in different health facilities is not satisfactory to the patients (11–13). The patient-centered communication behavior of the health care providers which plays the most vital role in patient satisfaction is often overlooked in this country (14). It was reported that almost one-third of the patients were dissatisfied with the healthcare service they receive especially those who attend public primary care facilities (13, 15). The quality of communication with the health care provider and the environment of the facility plays the most vital role in this regard (13). However, these scattered pieces of evidence mostly reflect the situation of the outpatient service of the health care facilities of the country as the majority of the study was conducted in outpatient settings. There is hardly any evidence regarding the satisfaction level of the patients admitted to the inpatient department of different public and private healthcare facilities in the country. Hence, the objective of this study was to investigate the level of satisfaction and its associated factors among admitted patients in tertiary care public and private hospitals in Bangladesh.

## Methods

### Study design and setting

A facility-based cross-sectional study was conducted in December 2019 in Dhaka Medical College Hospital (DMCH), Ibn Sina Hospital (ISH), and Crescent Hospital. Among these, DMCH is a 2000-bedded government tertiary care teaching hospital situated in Dhaka, the capital of Bangladesh. It serves as one of the major referral centers of the country with different super-specialized facilities. On the other hand, ISH and Crescent Hospital are two of the largest tertiary care private hospitals in the country equipped to deal with both conservative and operative procedures with proper diagnostics amenities.

### Study participants

Patients admitted to different medical and surgical facilities of the selected hospitals were considered as the study population. The sample size was calculated from the following formula: $n=\frac{z^{2}p\left(1-p\right)}{d^{2}}$, where, *z* = *z-*value for 95% confidence level, p = prevalence of patients' satisfaction and d = precision of error. Assuming the prevalence of patients' satisfaction as 63%, evidenced in a previous study (13), for 5% precision of error, the calculated sample size was 358. Being a multi-center study, we considered a design effect of 1.5 for the variance of the sample which provided the sample size as 537, rounded to 540 from each public and private facility (a total number of 1,080).

Inclusion criteria of the participants included adult patients (aged >18 years) of either sex admitted to the medical or surgical departments of the selected hospitals and stayed for at least 24 h. The pediatric patients, severely ill patients unable to participate in the survey, and those who were discharged within 24 h of admission were excluded. Consecutive patients admitted to the department of medicine and surgery of the selected hospitals meeting the inclusion and exclusion criteria were included in the study.

### Data collection tool and procedure

Patients' data were collected using a semi-structured questionnaire prepared based on existing evidence (4, 16–19). The primary draft of the questionnaire was prepared in English and then translated to Bangla through the back-translation method. It was then pre-tested among ten admitted patients in Sir Salimullah Medical College Hospital, Dhaka, a tertiary care hospital that was not included in this study.

The questionnaire had two parts:

**(i) Demographics and clinical characteristics of the patients:** This part included patients' socio-demographic information like age, sex, economic status, co-morbidity profile, department of admission, mode of treatment (conservative or surgical), duration of stay, perceived outcome of treatment and treatment cost. Treatment cost included admission fees, in-hospital accommodation, and food expenses, consultation fees, diagnostic expenses, instrumental and drug expenses, operative, interventional, and procedural fees which was the out-of-pocket expenditure by the patients. Patients' self-reported outcome was measured by a single item question during discharge to the patients or their attendants (if patients were unable) ‘How do you feel now compared to the time of admission?' with options of improved, not improved, and deteriorated.
**(ii) Patients' satisfaction:** The level of patient satisfaction was measured using a questionnaire adapted from the Brief Emergency Department Patient Satisfaction Scale (BEDPS) which was modified to assess patient satisfaction with the services provided in the inpatient department (19). The scale was divided into five parts containing a total of 20 questions (6 questions related to staff, 3 questions about the inpatient environment, 4 questions about patient care satisfaction, 5 questions about general patient satisfaction, and 2 regarding patients' family satisfaction). The questions were scored according to a Likert scale graded as Very dissatisfied (1), Dissatisfied (2), Fair/indifferent (3), Satisfied (4), and Very satisfied (5). A score equal to or below the mean was considered as dissatisfied while a score above the mean was labeled as satisfied (16). The questionnaire showed acceptable internal consistency (Cronbach's alpha 0.87).

A face-to-face interview was conducted with the patient by trained data collectors for obtaining patients' information. For quality control of the collected data, extensive training was provided to data collectors. The cross interview was conducted with almost 10% of the participants for data accuracy, and no difference was found in the outcome of double-entered data. Data collectors were not the hospital staff and did not wear hospital uniforms to avoid respondent bias. Collected data was checked for completeness every day by the research assistant team before data entry.

Participants' private information wasn't gathered, examined, or kept either during or after the study. Information about each participant's participation and accomplishments was kept secret. The results of the experiment had no effect on the health of patients and treatment process as this study did not collect any data regarding their treatment process, medicine or mental health. Data on participants' satisfaction were identified using special unique identification numbers that were randomly generated for each participant at the beginning of the study.

### Ethics statement

All the participants were enrolled in the study upon informed written consent. They were notified about the purpose of the study, the right to refuse to participate in the study, and the confidentiality of the information gathered. Ethical approval of the study proposal was obtained from the Institutional Review Board of the University of Rajshahi.

### Statistical analysis

The STATA version 17 was used for statistical analyses. All the paper-based data were cleaned, coded, and entered into the STATA software. Descriptive statistics were used to present the socio-demographic characteristics and disease profiles of the patients and their satisfaction levels. The chi-square test and multiple logistic regression models were used to identify factors affecting the patient satisfaction level. Three logistic regression models were constructed (model I including the treatment cost, model II including treatment cost and hospital type, and model III including these factors adjusted for patients' sociodemographic characteristics) to determine the factors associated with patients' satisfaction. Results of the analysis were presented as an adjusted odds ratio (aOR) with 95% CI.

## Result

### Sociodemographic characteristics

A total of 1,080 patients admitted to the inpatient department of the Medical and Surgical ward of the selected hospital were approached for an interview. After excluding the non-responding patients and incomplete data a finally 923 patients (420 from government and 503 from private hospitals) were included in the analysis. The average age of the included patients was 51 years (SD 20.1 years) and almost 60% of them were men. Almost half of the patients hailed from urban areas and middle-income families. Almost two-thirds of the patients were accommodated in general wards; however, usage of cabins was higher in private hospitals compared to government hospitals. Almost 23% of the patients admitted to government hospitals were accommodated on the floor. Almost 65% of the patients left the hospital within seven days of admission which was higher in private hospitals 75%). The majority of the patients (86%) received conservative treatment and reported improvement in their health condition after the treatment. The median treatment cost of the patients was BDT 12342 in government hospitals and BDT 41200 in private hospitals (Table 1).

**Table 1:** Sociodemographic and clinical characteristics of the patients (*n* = 923).

**Characteristics**	**Total**	**Government hospital**	**Private hospital**	**p-value**
Age (years) (mean, SD)	50.77 (20.06)	50.10 (21.10)	51.33 (19.15)	0.349
**Sex**				
Male	548 (59.37)	276 (65.71)	272 (54.08)	<0.001
Female	375 (40.63)	144 (34.29)	231 (45.92)	
**Residence**				
Urban	471 (51.03)	228 (54.29)	243 (48.31)	0.071
Rural	452 (48.97)	192 (45.71)	260 (51.69)	
**Occupation**				
Student	124 (13.43)	66 (15.71)	58 (11.53)	<0.001
White collar job	269 (29.14)	120 (28.57)	149 (29.62)	
Blue collar job	66 (7.15)	44 (10.48)	22 (4.37)	
Housemaker	229 (24.81)	78 (18.57)	151 (30.02)	
Retired/Unemployed	235 (25.46)	112 (26.67)	123 (24.45)	
**Family income**				
Low	247 (26.76)	212 (50.48)	35 (6.96)	<0.001
Middle	596 (64.57)	204 (48.57)	392 (77.93)	
High	80 (8.67)	4 (0.95)	76 (15.11)	
**Bed type**				
Cabin	258 (27.95)	28 (6.67)	230 (45.73)	<0.001
General bed	665 (72.05)	392 (93.33)	273 (54.27)	
**Duration of stay**				
≤ 7 days	603 (65.33)	226 (53.81)	377 (74.95)	<0.001
>7 days	320 (34.67)	194 (46.19)	126 (25.05)	
**Treatment**				
Conservative	793 (85.92)	310 (73.81)	483 (96.02)	<0.001
Surgical	130 (14.08)	110 (26.19)	20 (3.98)	
**Patient reported outcome**				
Improved	822 (89.06)	322 (76.67)	500 (99.40)	<0.001
Not improved/deteriorated	101 (10.94)	98 (23.33)	3 (0.60)	
Cost (BDT), median (IQR)	21,921 (32,750)	12,342 (8,782)	41,200 (43,556)	<0.001

### Patients' satisfaction

Almost 65% of the patients were satisfied with the inpatient care services they received. Regarding the domain specific categories, the patient satisfaction rates were 62.6% in hospital staff courtesy, 56.5% in a hospital environment, 67% in physician care, 63% in general patient satisfaction, and 58% in patient's family care (Table 2).

**Table 2:** The overall satisfaction rate of the admitted patients (*n* = 923).

**Questions**	**Satisfied (%)**	**Dissatisfied (%)**
**Staff courtesy**		
1. Nurses care about my treatment	587 (63.60)	336 (36.40)
2. Nurses inform me about the remaining of the treatment	542 (58.72)	381 (41.28)
3. Nurses attended to me patiently	601 (65.11)	322 (34.89)
4. Nurses relieved me of the pain well	555 (60.13)	368 (39.87)
5. Admission staff guided me appropriately	554 (60.02)	369 (39.98)
6. The behavior of the attending staff was suitable	587 (63.60)	336 (36.40)
**Environment**		
7. The environment was calm and quiet	520 (56.34)	403 (43.66)
8. Ward/Cabin was well equipped	510 (55.25)	413 (44.75)
9. The environment was hygienic	530 (57.42)	393 (42.58)
**Physician care satisfaction**
10. The physician told me about my treatment course	588 (63.71)	335 (36.29)
11. The behavior of the physician was respectful	580 (62.84)	343 (37.16)
12. The physician's explanation about the remaining of treatment was enough	644 (69.77)	279 (30.23)
13. The physician spent a sufficient time examining me	602 (65.22)	321 (34.78)
**General patient satisfaction (GPS)**		
14. The waiting time before seeing the doctor was appropriate	580 (62.84)	343 (37.16)
15. The waiting time before admission process was appropriate	569 (61.65)	354 (38.35)
16. I would recommend this hospital to my acquaintances	591 (64.03)	332 (35.97)
17. I am satisfied with the quality of services in the hospital	567 (61.43)	356 (38.57)
18. The hospital is well functioning	540 (58.50)	383 (41.50)
**Patient's family satisfaction**		
19. The family of the patient are respected in this hospital	587 (63.60)	336 (36.40)
20. Family can spend an appropriate amount of time besides the patient	610 (66.09)	313 (33.91)
**Overall satisfaction**		
The overall satisfaction of staff courtesy	577 (62.56)	346 (37.44)
The overall satisfaction of environment	521 (56.47)	402 (43.53)
The overall satisfaction of physician care	621 (67.24)	302 (32.76)
The overall score of general patient satisfaction	585 (63.36)	338 (36.64)
The overall satisfaction patient's family care	539 (58.45)	384 (41.55)
Total overall satisfaction level of the patients toward the ED services	599 (64.89)	324 (35.11)

### Factors associated with patients' satisfaction

In bivariate analysis, it was found that patients admitted to private hospitals, women, those who stayed <7 days, received conservative treatment, and reported perceived improvement in their health condition were more likely to be satisfied. On the contrary, patients' accommodation on the floor, surgical management, and higher treatment cost was associated with patients' dissatisfaction (Table 3). In subgroup analysis, predictors of the satisfaction subscale were similar to the overall satisfaction, hence data is not shown.

**Table 3:** Factors associated with satisfaction of the admitted patients (*n* = 923).

**Characteristics**	**Satisfied**	**Not satisfied**	***p*-value (Chi-square test)**
**Hospital type**			
Government	214 (50.95)	206 (49.05)	0.003
Private	385 (76.54)	118 (23.46)	
Age, Mean (SD)	52.10 (20.11)	50.53 (21.25)	0.675
**Sex**			
Male	341 (62.23)	207 (37.77)	0.041
Female	258 (68.80)	117 (31.20)	
**Residence**			
Urban	300 (63.69)	171 (36.31)	0.746
Rural	299 (66.15)	153 (33.85)	
**Bed type**			
Cabin	381 (66.96)	188 (33.04)	0.001
General bed	218 (61.58)	136 (38.42)	
**Duration of stay**			
≤ 7 days	412 (68.33)	191 (31.67)	0.001
>7 days	187 (58.44)	133 (41.56)	
**Occupation**			
Student	87 (70.16)	37 (29.84)	0.719
White collar job	168 (62.45)	101 (37.55)	
Blue collar job	43 (65.15)	23 (34.85)	
Housemaker	165 (72.05)	64 (27.95)	
Retired/Unemployed	136 (57.87)	99 (42.13)	
**Family income**			
Low	159 (64.37)	88 (35.63)	0.057
Middle	383 (64.26)	213 (35.74)	
High	57 (71.25)	23 (28.75)	
**Treatment**			
Conservative	560 (70.62)	233 (29.38)	0.012
Surgical	39 (30.00)	91 (70.00)	
**Patient reported outcome**			
Improved	548 (66.67)	274 (33.33)	0.001
Not improved/deteriorated	51 (50.50)	50 (49.50)	
Cost (BDT), median (IQR)	23,870 (34,220)	21,037 (28,517)	0.021

In multiple logistic regression models, higher treatment cost was associated with patients' dissatisfaction with the in-patient service they received in both public and private hospitals even after adjustment for their socio-demographic and clinical characteristics (aOR 0.97, 95% CI 0.95–0.99). However, patients admitted in private hospitals were more satisfied compared to government hospitals (aOR 3.64, 95% CI 2.2–6.03). Patients' accommodation on the floor was associated with dissatisfaction (aOR 0.38, aOR 0.22–0.66). Conservative management (aOR 3.34, 95% CI 2.10–5.33), shorter hospital stay (<7 days) (aOR 1.58, 95% CI 1.05–2.37), and perceived improvement after treatment (sOR 1.67, 95% CI 1.01–2.76) were also associated with patients' higher level of satisfaction (Table 4).

**Table 4:** Determinants of patients' satisfaction after admission (Logistic regression models).

**Factors**	**Model I aOR (95% CI)**	**Model II aOR (95% CI)**	**Model III aOR (95% CI)**
Treatment cost (per thousand BDT)	0.95 (0.92–0.98)	0.98 (0.97–0.99)	0.97 (0.95–0.99)
Private vs. Government^R^ hospital		3.14 (2.37–4.16)	3.64 (2.20–6.03)
Female vs. Male^R^ sex			1.07 (0.70–1.64)
Accommodation in general bed vs. Cabin^R^			0.38 (0.22–0.66)
≤ 7 vs. >7^R^ days of stay			1.58 (1.05–2.37)
Conservative vs. Surgical^R^ management			3.34 (2.10–5.33)
Perceived improvement vs. No improvement^R^			1.67 (1.01–2.76)

aOR, Adjusted odd's ratio; R, Reference category.

## Discussion

This study provided an overview of the satisfaction level with the service provided to the patients admitted to the selected tertiary care hospitals of Bangladesh. Here, almost 65% of patients were satisfied with the service they received. Moreover, patients admitted to private hospitals had a higher satisfaction level 76.5%) compared to those in government hospitals (51%). Almost 67% and 63% of the patients were satisfied with the care from physicians and hospital staff respectively and 56% were satisfied with the hospital environment.

There is little evidence on the satisfaction level of hospital-admitted patients in Bangladesh. A study conducted through outdoor patients' exit interviews reported that almost 63% of the patients were satisfied with the service provided (13). Other studies conducted in rural health facilities reported that almost 75% of patients were satisfied; however, this was also an outdoor-based study (20). Some other studies also reported average levels of patient satisfaction though these reported levels of satisfaction levels on a continuous scale which is hard to compare with the findings of this study (12, 15). However, one of these studies reported a similar satisfaction score in both private and public hospitals in contrast to our findings (12). A study from neighboring India reported a higher level of satisfaction with an overall satisfaction rate of almost 93% (21). However, this study was conducted in patients admitted to a private nursing home, where the quality of service might be better compared to the public hospitals. Satisfaction levels in different government hospitals ranged from 60 to 77% which is comparable to our findings (22, 23). A similar level of patient satisfaction was reported in other developing countries like Nepal (24), Pakistan (25), China (26), Fiji (6), Nigeria (18) and Ethiopia (4), etc.

We found treatment cost was a significant predictor of patients' satisfaction in both public and private facilities. Higher health care cost was identified as a predictor of patients' dissatisfaction in several studies before (27–30). However, it is a vicious cycle. It was reported that patient satisfaction often results from increased health care expenditure, increased hospital admission, and increased number of drugs in prescription (27). One possible cause of being high treatment costs associated with patients' dissatisfaction might be the lack of insurance coverage for health expenditure in Bangladesh (31). Health insurance coverage is found to have a positive impact on overall patient satisfaction levels as it minimizes the patients' concerns about healthcare costs and increases positive perceptions of the care providers (26, 30). However, despite higher healthcare costs, patients admitted to private hospitals reported a higher rate of satisfaction compared to public hospitals. A higher level of satisfaction was also reported in patients from private hospitals in other studies from both Bangladesh and other countries (11, 21, 24, 25). Studies reported that service quality, the environment of health care facilities, and responsiveness of care providers like physicians and nurses play a significant role in shaping patients' perception of the facility and their overall satisfaction (25–27). Private hospitals often attempt to provide a better experience to their patients considering the competitive marketplace. On the other hand, public hospitals have the responsibility to ensure health care for the mass population and remain burdened with an overwhelming number of patients and a limited number of care providers which constrains their ability to provide quality care for every patient. For example, in our study, almost 23% of patients in public hospitals were treated on the floor and it was associated with a high level of dissatisfaction. Hence, satisfaction level often falls among the patients in these hospitals.

Patients' treatment modality often affects their satisfaction level. In our study, patients receiving conservative management had a higher satisfaction rate compared to those who received surgical management. A similar finding was reported in a number of previous studies (16, 32, 33). However, it is an ambiguous phenomenon. Surgical management also reported a higher satisfaction rate in some cases (34). Treatment outcome has an influence in this regard. Patients with better treatment outcome and shorter hospital stay often report a higher satisfaction level as found in our study, as well as in previous ones (5, 27). Besides these hospitals and treatment-related factors, patients' sociodemographic characteristics are also associated with satisfaction levels. Older patients with previous experience of hospital visits as patients from lower socioeconomic conditions often reported a higher rate of satisfaction (4, 13, 16, 24). However, patients' education level showed an ambiguous relationship with satisfaction. Educated patients often have a high expectation level from the health care facilities which decreases their satisfaction level with the service they are provided. In contrast, these patients sometimes reported higher satisfaction levels considering the limitations of the hospitals, especially in public settings (5, 13, 16).

This study has several limitations. First of all, the satisfaction level is a perceived idea of the patients which is crafted through the interaction of their expectations, attitudes, and quality of service they receive. Hence, a qualitative exploration could provide a better picture of the influencing factors of their satisfaction level. Moreover, there might be some confounding variables that we did not include in the data collection tool that could have an effect on patient satisfaction, including patients' detailed sociodemographic characteristics, patients' overall disease profile, clinical presentation, and time of hospital admission. Moreover, the question used for patient-reported outcomes in our study was not validated. Finally, the study only included tertiary care hospitals; hence, findings might not be generalizable to all levels of health care facilities in the country.

## Conclusion

In summary, almost two-thirds of the patients admitted to tertiary care hospitals were satisfied with the inpatient service they received with a higher satisfaction rate in private hospitals. The lowest satisfaction level was found in the case of the hospital environment and patients' family care. Lower treatment cost, shorter hospital stay, conservative management, and perceived improvement after treatment were associated with the satisfaction level of the patients. More patient-centered care at the lowest possible cost, as well as improvement of the hospital environment, might increase the satisfaction level of the patients.

## Data availability statement

The raw data supporting the conclusions of this article will be made available by the authors, without undue reservation.

## Ethics statement

The studies involving human participants were reviewed and approved by Ethical Review Board of University of Rajshahi. The patients/participants provided their written informed consent to participate in this study.

## Author contributions

FB: conceptualization, study design, writing-original draft preparation, data collection, methodology, and formal analysis. JS, SH, and MA: editing, supervision, and reviewing. All authors have read and agreed to the published version of the approved manuscript.

## Conflict of interest

The authors declare that the research was conducted in the absence of any commercial or financial relationships that could be construed as a potential conflict of interest.

## Publisher's note

All claims expressed in this article are solely those of the authors and do not necessarily represent those of their affiliated organizations, or those of the publisher, the editors and the reviewers. Any product that may be evaluated in this article, or claim that may be made by its manufacturer, is not guaranteed or endorsed by the publisher.
